# Adventitious Shoot Regeneration from Leaf Explant of Dwarf Hygro (*Hygrophila polysperma* (Roxb.) T. Anderson)

**DOI:** 10.1155/2013/680425

**Published:** 2013-06-17

**Authors:** Mehmet Karataş, Muhammad Aasim, Ayşegül Çınar, Muhammet Dogan

**Affiliations:** Department of Biology, Kamil Ozdag Faculty of Science, Karamanoglu Mehmetbey University, Yunus Emre Campus, 70200 Karaman, Turkey

## Abstract

Dwarf hygro (*Hygrophila polysperma*) is an ornamental aquatic plant that changes its leaf colours to pinkish in high light. It is listed as a medicinal plant in medicinal plant lists of Indian states of West Bengal and Karnataka. It is also used as a screening tool for toxicities and a bioindicator to detect and control algae. The study reported *in vitro* adventitious shoot regeneration from leaf explants cultured on MS medium containing 0.10–1.60 mg/L Kin/TDZ with or without 0.10 mg/L IBA and 500 mg/L Amoklavin to eradicate endogenic bacterial contamination. Direct adventitious shoot regeneration started within one week from both culture mediums followed by late callus induction which was more prominent on TDZ containing media compared to Kin containing media. Addition of 0.10 mg/L IBA with both Kin and TDZ increased shoot regeneration frequency, mean number of shoots per explant, and mean shoot length. Maximum number of 16.33 and 20.55 shoots per explant was obtained on MS medium containing 0.80 + 0.10 mg/L Kin-IBA and 0.10 + 0.10 mg/L TDZ-IBA, respectively. Regenerated shoots were rooted on MS medium containing 0.20–1.00 mg/L IBA followed by successfull acclimatization in aquariums. Regenerated plantlets were also tested in jars containing distilled water that showed the pH 6–9 for the best plant growth and development.

## 1. Introduction

Aquatic plants are partially or completely water grown plants, and they are gaining popularity in traditional aquariums and water gardens [1]. In USA, water gardens are found in approximately 16 million houses [2]. In European countries, aquatic plants are also popular and are imported from abroad due to ever increasing popularity. Holland, France, Czech Republic, Germany, Hungary, Switzerland, Austria, Turkey, Latvia, and Estonia are the leading countries of Europe spending millions of Euros for the import of aquatic plants. The most imported aquatic plant is *Egeria densa* which is followed by *Cabomba caroliniana, Hygrophila polysperma, Vallisneria spiralis, Echinodorus bleheri, Vallisneria americana, Najas marina, *and* Hygrophila difformis *[3]. 

*Hygrophila Polysperma* (Roxb.) T. Anderson, commonly known as dwarf hygrophila, dwarf hygro, Miramar weed, or Indian weed, is an aquatic plant belonging to family the Acanthaceae. Dwarf Hygro is native to India and Malaysia and was introduced to USA states of Texas, Florida, and Virginia [4] and traded as Eastern Ludwigia in 1945 [5]. The plant is very popular aquatic ornamental plant [6] and became a part of aquariums all over the world. Dwarf hygro belongs to *Hygrophila *genus which contains almost 90 species; most of them are used for medicinal [7, 8] and antibacterial [9] purposes. 

Dwarf hygro is an important ingredient of Ayurvedic system of medicine used for hemiplegia, stiff-neck, facial paralysis, and noise in the ears with headache [10]. The seeds of dwarf hygro are also used for treatment of other remedies in India [11], and the plant has been listed in the medicinal plant lists of Indian states of West Bengal [12] and Karnataka [13]. The research carried out in Sweden showed the efficient use of dwarf hygro to reduce the toxicity level [14]. Similarly, the plant has been used as bioindicator for algae control along with Indian ferns [15]. Very little is known about the importance of dwarf hygro micropropagation; therefore, the present study was designed to get adventitious shoot regeneration under *in vitro* conditions. Thereafter, the protocol can be employed to isolate secondary metabolites from the important medicinal plant. 

## 2. Material and Methods

The *H. polysperma* plants were obtained from local aquarium traders of Karaman province of Turkey. The plants were confirmed by Professor Dr. Hasan Huseyin Atar of the Department of Fisheries of the Ankara University, Turkey. Four-five cm long twigs containing 5-6 nodes with attached leaves were first washed for 5 min under tap water. Thereafter, they were surface sterilized with 24% H_2_O_2_ (40% v/v) for 10 min followed by 3 × 5 min rinsing with sterilized distilled water by continuous stirring. The leaves were separated from twigs under sterile conditions, cultured on MS [16] medium for 2 weeks to obtain contamination free explants.

Leaf explants were cultured on MS medium supplemented with 3% sucrose and 0.10–1.60 mg/L Kin-0, 0.10 mg/L IBA (Table 1) or 0.10–1.60 mg/L TDZ-0, 0.10 mg/L IBA (Table 2) in Magenta GA^7^ vessels solidified with 0.65% agar. Culture media were also supplemented with 500 mg/L Amoklavin (antibiotic) to eradicate bacterial contamination, if any. Each experimental treatment was run in hexaplate and contained 8 explant (8 × 6 = 48 explants) with the pH of all media adjusted to 5.8 ± 0.1 before autoclaving (118 kPa atmospheric pressure, 120°C for 21 min). Both shoot and root regeneration experiments were repeated twice. All cultures were incubated under 16 h light photoperiod (5000 lux) using white Fluorescent lights. 

The regenerated shoots were cultured on MS medium containing 0.10–1.00 mg/L IBA for rooting. After four weeks of culture, agar was removed carefully from the rooted plantlets without damaging the roots by washing under running tap water. Thereafter, the plants were transferred to aquariums containing tap water and sand. In another experiment, the plants were acclimatized in jars containing water at variable pH of 4.0–10.0 and then left open for for acclimatization in growth room at 23°C with 16 h light photoperiod for 3 weeks. 

All data shown in percentages were subjected to arcsine transformation [17] before statistical analysis. Statistical analysis was performed as one way ANOVA using SPSS17 for Windows, and post hoc tests were performed using LSD or *t*-test. 

## 3. Results

**Figure 1:**
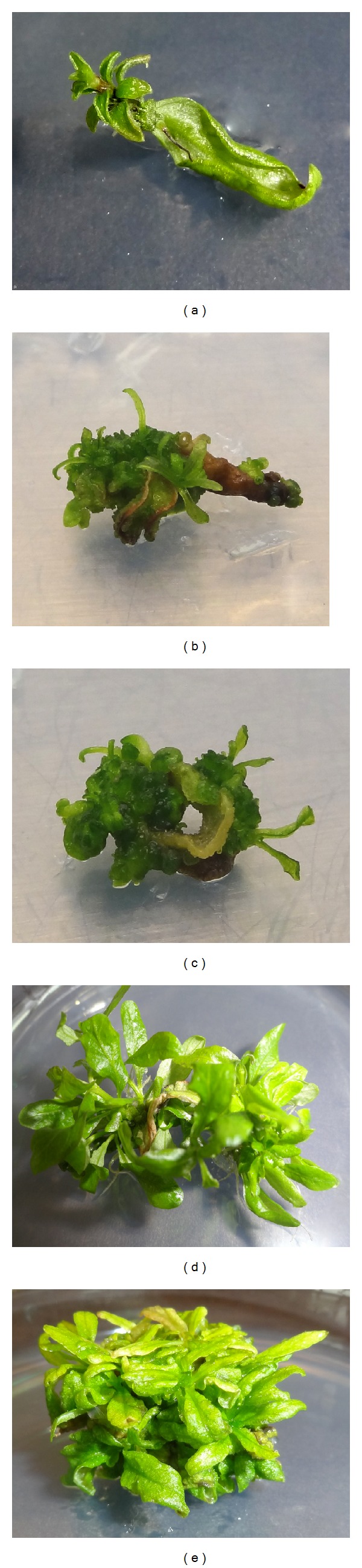
*In vitro* adventitious shoot regeneration of *H. polysperma:* (a) shoot induction from leaf tip, (b) multiple shoot initiation, (c) callus induction, and ((d) and (e)) multiple induced shoots.

Leaf explants of *H. polysperma* were cultured on MS medium containing 0.10–1.60 mg/L Kin or TDZ with or without 0.10 mg/L IBA. Direct adventitious shoot regeneration without callus induction started from leaf tip on both Kin-IBA and TDZ-IBA containing basal media. After one week of culture, visible shoot buds were noted on the leaf tips (Figure 1(a)) and the margins (Figure 1(b)) of the explants. It was followed by callus induction (Figure 1(c)) and induction of multiple shoots on MS medium containing Kin-IBA (Figure 1(d)) and TDZ-IBA (Figure 1(e)). 

Callus induction (Figure 1(c)) started after 3-4 weeks of culture on both Kin-IBA and TDZ-IBA supplemented medium. However, callus induction from TDZ-IBA was earlier compared to Kin-IBA. Frequency of callus induction (%) on MS medium supplemented with Kin-IBA and TDZ-IBA ranged 0.00%–75.00% (Table 1) and 50.00%–100.00% (Table 2), respectively. Very low or marginal frequency of callus induction was recorded on Kin used singly. However, TDZ used singly induced more callus on leaf explants ranged 50.00%–93.75%. Addition of 0.10 mg/L IBA positively induced callus with both Kin and TDZ in the culture medium that ranged 50.00–75.00% (Table 1) and 81.25–100.00% (Table 2) on MS medium containing Kin-IBA and TDZ-IBA, respectively. 

Comparing shoot regeneration frequency (%), it varied on both regeneration media with range of 0.00%–100.00% on MS medium containing Kin-IBA and 62.50%–100.00% on MS medium containing TDZ-IBA. Both Kin and TDZ without IBA induced lower shoot regeneration in ranges of 0.00%–50.00% and 62.50%–100.00%, respectively. However, inclusion of 0.10 mg/L IBA with Ki or TDZ was favorable and increased the shoot regeneration significantly to 91.67%–100.00% (Table 1) with Kin and 100.00% on all concentrations of TDZ (Table 2). 

Mean number of shoots per explants from MS medium supplemented with Kin singly yielded very low number of shoots (0.00–1.83) per explant (Table 1). Contrarily, TDZ singly induced more number of shoots per explant in range of 9.40–13.67 (Table 2). On the other hand, addition of 0.10 mg/L IBA with both Kin and TDZ positively increased the mean number of shoots per explant. Kin-IBA containing medium yielded 2.41–16.33 shoots per explants with maximum of 16.33 shoots obtained on 0.80 mg/L Kin-0.10 mg/L IBA (Table 1). Number of shoots per explant on TDZ-IBA containing MS medium ranged 13.43–20.55 per explant with maximum number of shoots induced on MS medium containing 0.10 mg/L TDZ-0.10 mg/L IBA (Table 2). 

Relatively longer shoots were obtained on MS medium containing Kin-IBA compared to MS medium containing TDZ-IBA. Increase of both Kin and TDZ concentrations in the culture media alone or with 0.10 mg/L IBA hindered mean shoot length. However, addition of 0.10 mg/L IBA improved the mean shoot length when culture contained various concentrations of Kin and TDZ singly. Mean shoot length of Kin-IBA and TDZ-IBA containing media ranged 0.00–1.15 cm (Table 1) and 0.31–0.86 cm (Table 2), respectively. However, maximum shoot length on both growth variants in their own category was recorded on MS medium supplemented with 0.10 mg/L Kin/TDZ + 0.10 mg/L IBA. 

**Figure 2:**
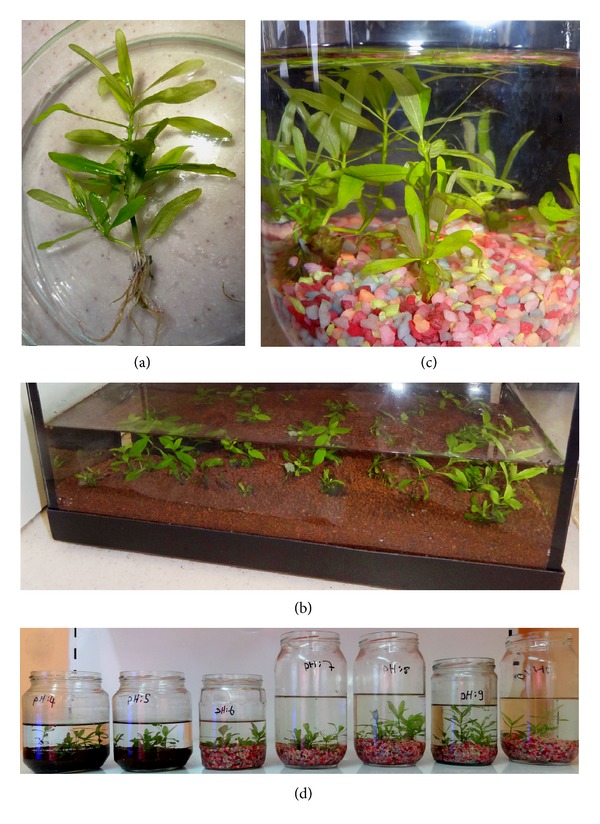
Rooting and acclimatization of *in vitro* regenerated plants of *H. polysperma*: (a) rooted plantlets, (b) acclimatized plants in aquariums, and (c) plant acclimatization at different pH.

Well-developed *in vitro* regenerated shoots were rooted on MS medium containing 0.10–1.00 mg/L IBA. 100.00% rooting was recorded after 4 weeks of culture. The rooted plants (Figure 2(a)) were successfully transferred to aquariums (Figure 2(b)) containing tap water and sand, where the plants achieved 100.00% survival and acclimatization after 2 months. 

*In vitro* rooted plantlets were transferred to glass jars containing distilled water at pH of 4.0–10.0 (Table 3). The experiment was run in triplicate with 5 plants selected randomly and placed in the jar. The plant height and number of internodes of all plants were measured before transfer to jars. After three weeks of culture, the plants were reassessed for plant height and number of internodes. All plants in the experiment showed 100% survival at all pH levels (Figures 2(c) and 2(d)) with visible effects on plant height and internodes. After three weeks, an increase of 9.86%–74.21% (Table 3) in plant height was recorded, with minimum increase of 9.86% on cultures at pH 4.0 followed by 26.11% increase at pH 5.0. However, maximum increase of 74.21% in plant height was recorded at pH 7.0. On the other hand, minimum of 30.76% and 35.71% increase in number of internodes was recorded (Table 3) at pH 10.0 and 4.0, respectively. 

## 4. Discussion 

The study presents the first report of *in vitro* adventitious shoot regeneration from leaf explant of *H. polysperma. *Leaf explant has been used for adventitious shoot regeneration in other aquatic plants like *Nymphaea* [18], *Hygrophila auriculata* [19]*, Rotala macrandra *[20], and* B. monnieri* [21]. Leaf explants responded well to both growth variants (Kin and TDZ). Direct adventitious shoot regeneration without callus induction was observed within two weeks from both culture medium used in the experiment. Direct shoot regeneration from leaf explant has been reported in *Spilanthes acmella *[22]. Shoot bud initiation from tips or edges showed the efficacy of leaf explant which might be due to presence of relatively younger and actively dividing cells in that zone. Valobra and James [23] also reported adventitious shoot regeneration from callus induced on leaf disc edges. 

Callus induction by the use of cytokinins-auxins concentrations is a reported phenomenon, and callus induction from leaf explant has been reported in other aquatic plants like water lettuce [24] and *B. monnieri* [21]. Callus induction started late after 3-4 weeks of culture in this study. Likewise, late callus induction from seed explant has been reported by Aasim et al. [25] in hairy vetch and Javed et al. [26] in *Brassica napus.* It was also noted that growth variants in the culture medium were more supportive for shoot induction rather than callus induction. Results further showed that frequency of callus induction was affected by type and concentration of growth variants and presence or absence of IBA in the culture medium. In general, TDZ used singly induced more calluses compared to Kin used singly. TDZ results in callus induction better than other cytokinins [27]. TDZ-induced callus induction on different explants of many recalcitrant species as well as on medicinal plants has been reported [28]. Contrarily, Başalma et al. [29] reported suppression of callus formation by TDZ in *Astragalus cicer* hypocotyl and cotyledon explants. On the other hand, MS medium containing Kin or TDZ with IBA induced more calluses compared to MS medium containing Kin or TDZ without IBA. Similarly, Mirici [30] reported low callus formation from leaf explants in *Astragalus polemoniacus* cultured on a medium with TDZ used singly. 

Results on shoot regeneration frequency also showed clear effects of type of growth variants. Comparing Kin and TDZ used singly, TDZ was more inductive compared to Kin. Vijayakumar et al. [31] reported 30.0%–95.0% and 50.0%–95.0% shoot regeneration frequency of *B. monnieri* cultured on BA and TDZ, respectively. On the other hand, both Kin and TDZ were very responsive in the presence of 0.10 mg/L IBA. Karatas et al. [21] reported 100.0% shoot regeneration on leaf explant of *B. monnieri* using various concentrations of BA-NAA. Contrarily, Aasim et al. [25] found insignificant effect of IBA on shoot regeneration frequency of hairy vetch. 

Results on mean number of shoots per explants revealed the effects of growth regulator type, concentration and presence or absence of auxins in the mediums. Results showed that Kin used singly is not sufficient for shoot buds initiation with very low number of shoots per explant compared to TDZ used singly. Contrarily, Yenice [32] reported 57.82 and 50.74 plantlets per explant of *Lemna minor* on liquid MS medium containing with 0.05 mg/L Kin and 0.6 mg/L TDZ, respectively. However, provision of 0.10 mg/L IBA proved to be sufficient for multiple shoot regeneration. Combination of cytokinin + auxin irrespective of their concentrations and explant type has been reported for maximum number of shoots per explant in other aquatic plants. Öztürk [33] obtained maximum number of shoots per explants on apical meristem explant of ludwiga cultured on 0.05 mg/L TDZ + 0.1 mg/L NAA. Anthony et al. [34] also recorded maximum number of shoots from *Leucopogon verticillatus* on 10 *μ*M TDZ + 5 *μ*M IAA. Panigrahi et al. [19] recorded maximum number of shoots on MS medium containing with 2 mg/L BAP and 0.2 mg/L NAA in *Hygrophila auriculata. *Öztürk [35] recorded 80.56 shoots per explant from leaf explant of *H. difformis* cultured on MS medium containing 0.25 mg/L Kin and 1 mg/L NAA. Sumlu [20] reported 27.33 shoots per explant of *Rotala macrandra *cultured on liquid MS medium containing 0.25 mg/L BAP + 0.50 mg/L NAA. 

Results on mean shoot length showed suppressive effects of TDZ compared to Kin used singly. Results further showed that increase in Kin/TDZ concentration resulted in stunted shoots; the results are in line with Lata et al. [36]. The suppressive effects of TDZ on shoot length might be consistent with its high cytokinin activity [37]. On the other hand, addition of IBA helped to overcome the negative effects of cytokinins; a has been reported in hairy vetch [25], fenugreek [38] and narbon vetch [39]. 

Regenerated shoots were successfully rooted using IBA in line with the findings of Tiwari et al. [40], Sharma et al. [41], and Karatas et al., [21]. Rooted plantlets were successfully acclamatized in aquariums which is an important step for *in vitro* regenerated plants and has been reported in other aquatic plants like *R. macrandra* [20], *L. repens *[33], *N. indica* [42], *A. sessilis* [43], *V. anagallis-aquatica* [44], *C. wendtii *and* C. beckettii* [45], and *B. monnieri *[21, 46]. 

In order to find out the most suitable pH level for acclimatization, the rooted plants were acclimatized directly under aquatic conditions at various pH levels. The results showed that a plant can survive at pH 4.0 to 10.0. The results are in line with the findings of Karatas et al. [21], who also reported no negative effects of pH levels and successfully acclimatized plants at pH 4.0–10.0. Results further showed that a plant showed slower growth at pH 4.0 and 10.0. and can grow vigorously at pH range of 6.0–9.0. 

## Figures and Tables

**Table 1:** Effects of different Kin-IBA concentrations on shoot regeneration of *H. polysperma *from leaf explant.

Kin (mg/L)	IBA (mg/L)	Callus induction (%)	Shoot regeneration (%)	Shoots per explant	Shoot length (cm)
0.10	—	0.00^b^	0.00^c^	0.00^e^	0.00^c^
0.20	—	0.00^b^	33.33^b^	2.53^d^	0.70^ab^
0.40	—	0.00^b^	50.00^b^	1.83^d^	0.47^bc^
0.80	—	8.33^b^	41.67^b^	1.83^d^	0.42^bc^
1.60	—	8.33^b^	41.67^b^	1.33^de^	0.45^bc^
0.10	0.10	50.00^a^	100.00^a^	2.41^d^	1.15^a^
0.20	0.10	75.00^a^	91.67^a^	8.66^c^	0.76^ab^
0.40	0.10	75.00^a^	91.67^a^	11.20^b^	0.78^ab^
0.80	0.10	66.66^a^	100.00^a^	16.33^a^	1.03^ab^
1.60	0.10	75.00^a^	91.67^a^	12.58^b^	0.82^ab^

Means followed by different small letters within columns are significantly different using Duncan *P* < 0.05.

**Table 2:** Effects of different TDZ-IBA concentrations on shoot regeneration of *H. polysperma *from leaf explant.

TDZ (mg/L)	IBA (mg/L)	Callus induction (%)	Shoot regeneration (%)	Shoots per explant	Shoot length (cm)
0.10	—	75.00^a^	87.50^ab^	10.18^bc^	0.43^c^
0.20	—	93.75^a^	100.00^a^	12.68^abc^	0.35^c^
0.40	—	75.00^a^	75.00^bc^	11.95^abc^	0.36^c^
0.80	—	50.00^b^	62.50^c^	9.40^c^	0.36^c^
1.60	—	87.50^a^	87.50^ab^	13.67^abc^	0.38^c^
0.10	0.10	81.25^a^	100.00^a^	20.55^a^	0.86^a^
0.20	0.10	100.00^a^	100.00^a^	19.61^a^	0.72^b^
0.40	0.10	100.00^a^	100.00^a^	13.43^abc^	0.38^c^
0.80	0.10	100.00^a^	100.00^a^	18.43^ab^	0.34^c^
1.60	0.10	100.00^a^	100.00^a^	15.52^abc^	0.31^c^

Means followed by different small letters within columns are significantly different using Duncan *P* < 0.05.

**Table 3:** Effects of pH levels on plant height and number of internodes of *H. polysperma*.

pH	Plant height before the start of experiment (cm)	Plant height after 3 weeks (cm)	Change in plant height (%)	Number of internodes	Number of internodes after 3 weeks	Change in number of internodes (%)
4.0	3.75	4.12	9.86	3.50	4.75	35.71
5.0	3.37	4.25	26.11	3.00	4.75	58.33
6.0	2.75	4.25	54.54	2.25	4.50	100.00
7.0	2.87	5.00	74.21	2.75	4.75	72.72
8.0	3.37	5.12	51.92	2.75	4.75	72.72
9.0	3.62	6.00	65.74	2.75	5.25	90.90
10.0	3.37	4.87	44.51	3.25	4.25	30.76
